# Characteristics of private partners in Chiranjeevi Yojana, a public-private-partnership to promote institutional births in Gujarat, India – Lessons for universal health coverage

**DOI:** 10.1371/journal.pone.0185739

**Published:** 2017-10-17

**Authors:** Veena Iyer, Kristi Sidney, Rajesh Mehta, Dileep Mavalankar, Ayesha De Costa

**Affiliations:** 1 Indian Institute of Public Health, Gandhinagar, Gujarat, India; 2 Department of Public Health Sciences, Karolinska Institutet, Stockholm, Sweden; 3 Department of Preventive and Social Medicine, Valsad Medical College, Valsad, Gujarat, India; National Academy of Medical Sciences, NEPAL

## Abstract

**Background:**

The Chiranjeevi Yojana (CY) is a Public-Private-Partnership between the state and private obstetricians in Gujarat, India, since 2007. The state pays for institutional births of the most vulnerable households (below-poverty-line and tribal) in private hospitals. An innovative remuneration package has been designed to disincentivise unnecessary cesareans. This study examines characteristics of private facilities which participated in the program.

**Methods:**

We conducted a cross-sectional survey of all facilities which had conducted any births between June 2012 and April 2013 in three districts. We identified 111 private and 47 public facilities. Ninety of the 111 private facilities did caesarean sections in the last three months and were eligible to participate in the CY program. Of these, 40 (44%) participated in the CY program. We conducted descriptive and bivariate analyses followed by a Poisson regression model to estimate prevalence ratios of facility characteristics that predicted participation.

**Results:**

We found that facilities participating in the CY program had a significantly higher likelihood of being general facilities (PR 1.9, 95% CI 1.3–2.9), or conducting lower proportion of cesarean births (PR 2.1, 95% CI 1.2–3.5) or having obstetricians new in private practice (PR 1.9, 95% CI 1.2–3.1) or being less expensive (PR 1.8, 95% CI 1.1–3.0). But none of these factors retained significance in a multi variable model.

**Conclusion:**

Private obstetricians who participate in the CY program tend to be new to private practice, provide general services, conduct fewer caesareans and are also less expensive. This is advantageous to the PPP and widens the target beneficiary groups that can be serviced by the PPP. The state should design remuneration packages with the aim of attracting relatively new obstetricians to set up practices in more remote areas. It is possible that the CY remuneration package design is effective in keeping caesarean rates in check, and needs to be studied further.

## Introduction

Globally, Maternal Mortality Ratio (MMR) has declined by 45%, from 380 to 210 per 100,000 births between 1990 and 2013, the period of the Millennium Development Goals. But it still stands at 190 and 510 in LMICs of South Asia and Sub-Saharan Africa respectively [1]. There is now a global commitment to reduce the maternal mortality ratio to less than 70 per 100,000 live births in every country by 2030 as part of the sustainable development goals [2].

The unpredictability of most direct obstetric complications which usually arise as emergencies during childbirth contributes to a large proportion of maternal deaths [3,4]. The presence of Skilled Birth Attendants, who are trained to perform signal functions for emergency obstetric care (EmOC) at the time of childbirth, is key to reducing maternal mortality [5,6]. It is recognized that all the 7 basic EmOC (BEmOC) functions (Injectable Antibiotics, Injectable Uterotonics, Injectable Anticonvulsants, Manual removal of placenta, Removal of retained products, Assisted Vaginal delivery, Neonatal resuscitation) and 2 comprehensive EmOC (CEmOC) functions (Caesarean section and Blood transfusions) require incremental levels of training and skill. Some and/or all of these functions may be performed by a physician, a nurse or a midwife, although all of them are grouped under the broad term of Skilled Birth Attendants (SBAs). An appropriate enabling environment, often a health facility, would ensure that these SBAs can perform effectively [7]. Therefore, the management of childbirths by SBAs in facilities has been advocated as a key strategy to reduce maternal and perinatal deaths [5,6].

As a result, in order to reduce maternal and perinatal deaths, a number of lower middle-income countries (LMICs) have promoted institutional births both in public and private facilities over the last few decades [8]. The use of the private sector for obstetric care has increased substantially all over the world. The World Bank estimates that the formal and informal private sectors have provided obstetric services for more than half of all births in South Asia and Sub-Saharan Africa [9,10]. In five South and South-east Asian countries, private provision was responsible for almost the entire increase in institutional births in the last two decades [11]. The provision of obstetric care by SBAs in facilities, and the consequent possibility of a reduction in maternal mortality is increasingly happening through the private sector.

However, this reduction has mostly happened among wealthier households and among educated women as these women are more likely to have the resources to pay the out-of-pocket costs that private care often requires [12]. Neglected populations in developing countries are unable to access and utilize maternal health care services due to socio-economic deprivation, geographical attributes and low literacy levels [13]. Improving maternal health outcomes can only be possible if health inequalities of these disadvantaged women are addressed in health interventions and policies [14].

It is expected that the inclusion of Universal Health Coverage (UHC) as a Sustainable Development Goal will address such disparities in the future by accelerating equity in access to quality health services [15]. Public-Private-Partnerships (PPPs) between the public and private health sector is one of the essential strategies to attain UHC [16], especially so in regions where private providers supply the bulk of health services as in the case of childbirth services in South Asia and Sub-Saharan Africa [9,10].

There have been numerous PPPs in LMICs in the 90s. But most of these have been in the area of sexual and reproductive health. PPPs for childbirth services have been few [17]. Voucher schemes for safe childbirth through private partners have been implemented in Uganda, Kenya, Cambodia, Bangladesh, Nepal and Pakistan [18,19]. Although some of these have been classified as large scale programs (outlay more than $ 1 million/year) they have been implemented in populations of 1.5 to 5 million, partnered with less than 50 private partners [20] and have not been evaluated for the attributes of private providers. In India, where 70% of all health care expenditure is made in the private sector [21], the recent National Health Policy has recommended the exploration of PPPs as one of the means towards Universal Health Coverage [22]. However, none of the state-wide PPPs for childbirth services which were implemented in Delhi, Haryana, Uttarakhand, Rajasthan and Kolkata were evaluated to elucidate provider characteristics that predict participation in a PPP. This is probably because these PPPs were on a small to medium scale (annual budget of less than $1 million) when compared to the Chiranjeevi (long-life) Yojana program (CY) of Gujarat state [20]. The CY program with an annual budget of more than $1 million, covered 40% of the state’s population (24 million eligible vulnerable population) and has lasted longer than a decade. At its pinnacle, the program partnered with one-third of the private providers in the state (865/2000) [23].

Gujarat, is the western-most state of India with a population of 60.4 million. Fifty-seven percent of the population is rural, 15% belong to Scheduled Tribes and 20% live below the poverty line [24]. The CY was a PPP designed in 2005 by the state of Gujarat in India, as a counter measure to the low availability of emergency obstetric care in the public sector; only eight obstetricians served in public sub-district level hospitals in rural areas [25]. The department of health invited specialist post-graduate qualified obstetricians practicing in the private sector to partner in the CY program if they possessed functioning in-patient facilities with (at least) 15 beds, labor and operating rooms, the ability to manage complicated births, perform caesarean sections and arrange for blood transfusions. Eight hundred and sixty-five obstetricians enrolled into the CY program in 2006–7. Each obstetrician was paid a fixed lump sum of 3600 USD (raised to 4800 USD during our survey) for childbirth services provided to every 100 vulnerable women belonging to below-poverty-line or Scheduled Tribe households (BPL/ST). The poverty line and scheduled tribe criteria for vulnerability which were used for targeting the beneficiaries of the CY program have been defined in the Indian constitution and are regularly updated based on planned and diverse sample surveys conducted across the nation. The calculation of the payment package for the CY program was made on the assumption that the 100 births would include 85 uncomplicated vaginal births, 8 complicated births and 7 caesarean sections [26]. This removed any monetary incentive for private partners to do unnecessary cesarean sections. More than a million births have already occurred under this program between 2005 and 2015. Participation has varied from a high of 865 to a low of 360 private obstetricians over this decade [27].

In order to be able to engage private actors in the pursuit of Universal Health Coverage, we need to know how many and which private providers might be willing to engage in such partnerships [28]. There are no studies reporting on the characteristics of private obstetricians who partner with the government for a public health program geared towards improving maternal health outcomes. This paper aims to study the characteristics of eligible private obstetricians who chose to enter into a partnership with the state government to increase access to intrapartum care to disadvantaged women under the CY PPP program.

## Methods

Ethical approval for this study was obtained from institutional review board at Indian Institute of Public Health Gandhinagar, Gujarat, India (ethical approval number: TRC-IEC No. 23/2012).

### Study area

Three heterogeneous districts from the western, central and eastern belts of the state, Sabarkantha, Surendranagar and Dahod, each with an average population of 2 million were selected. Each district is further sub divided into10 sub-districts each with a population of 100 000 to 200 000. These districts were purposively selected to represent varying geographic areas [29] socio-demographic indicators, and CY uptake in the state (Table 1). Together, these three districts had a considerably higher proportion of vulnerable subpopulations, defined here as being those living below poverty line or belonging to scheduled tribes. Nearly 60% of the population would be ‘vulnerable’ using this definition and therefore eligible for the CY benefit, but only 21% had received it till 2011. All three districts ranked among the lowest third of the state’s 26 districts on the human development indices.

**Table 1:** Profile of study districts.

	Population (in millions) [24]	Crude Birth Rate per 1000 [30]	% population rural [24]	% population Scheduled Tribe [24]	% population Below Poverty Line [30]	% eligible for CY benefit (BPL+ST) [24,31]	% of births conducted under Chiranjeevi in 2006–11 out of total registered [32]
Gujarat state	60.4	22.7	57.0	14.8	39.6	40.7	10.9
Sabarkantha dist	2.4	28.0	85.0	19.7	32.9	43.3	22.0
Surendranagar dist	1.7	23.0	72.0	0.9	46.5	45.4	10.1
Dahod dist	2.1	30.2	90.0	72.4	71.6	87.9	29.7
Total of 3 study districts	6.2	27.0	82.3	31	50.3	58.9	20.6

### Study design

A cross-sectional facility survey was conducted between June 2012 and April 2013 of all facilities which had provided any childbirth services in the past year in the three districts.

### Data collection

An initial master list of all public and private facilities which conducted any childbirth in the last one year was created from secondary data. We accessed data on public facilities from the website of the state’s department of health and private facilities from the state headquarters of the professional association of Obstetricians and Gynaecologists. All public and private facilities in this list were visited and occurrence of child births in the last one year was verbally verified. These initial facilities were asked to identify any further facilities in their neighborhood which may have conducted births in the past year. These were added to the list and again physically verified and further requested to identify more facilities. This method of snowballing was continued throughout the data collection period until no more new facilities could be identified.

All facilities which had provided any childbirth services in the past year were administered a modified version of the survey forms developed by the WHO, UNICEF, UNFPA and AMDD for the Monitoring emergency obstetric care handbook [33]. Sixteen surveyors and four supervisors were trained to administer questionnaires and carry out field supervision. The data used for this study was collected through two schedules, (i) The facility survey form which assessed facility characteristics, such as their location, years of functioning, bed strength, provision of services other than maternity, as well as information regarding referral services and routine patient care practices in the facility. This form included a section which had to be administered to the facility’s obstetrician regarding his/her age, years of experience, participation in the CY program, charges for a normal or caesarean delivery. There was also a section with a few items to be filled out by reviewing records such as number of vaginal, caesarean and complicated births in the last six months. (ii) The human resources form that recorded details of all staff working in each facility. Data collection for the elements that were used in this analysis was as follows;

1. Clerical or para-medical staff in each facility responded to questions pertaining to years of functioning of the facility, its bed strength and type (purely obstetric or combined with other specialties).
2. Obstetricians responded to the question regarding their completed years of experience as an obstetrician.
3. Facility records were reviewed for numbers and types of births over last six months.
4. Surveyors observed the availability of an obstetrician 24*7, confirmed the same through interviews with labor room nurses, and recorded this data as full-time or part-time.
5. Facilities that were eligible to participate in the CY program based on criteria set by the state [34] were tagged onto a GPS map.

### Definitions of variables used

1. Facility participation in the CY program: A CY participant facility was one that met the eligibility criteria set by the state [32] and was part of the CY program. A CY non-participant facility was one that met these criteria but was not a partner in the CY program at the time of the study.
2. Location of facilities: Private facilities could be located either in the three district headquarter towns (largest towns in the district) or in 18 out of 27 smaller sub-district headquarter towns in each of the three districts.
3. Facility Type: Facilities either provided purely maternity (and gynecological) in-patient care or they were general hospitals which provided out and inpatient care to men and children with or without the presence of specialists like a general physician, pediatrician, orthopaedician or surgeon.
4. Bed Strength: This indicated the number of beds in a facility. The state prescribes a preferable bed strength of ‘approximately’ 15 for CY participation, though actual bed strengths of facilities vary.
5. Obstetrician’s years of experience: Obstetricians reported the number of years they had been in practice since they completed their post-graduate training.
6. Average number of vaginal births performed over last six months by each facility: Surveyors reviewed facility records and documented the number of normal vaginal births each of the facilities had conducted during the six months before the survey. This was averaged to provide the mean number of births each month.
7. Proportion of caesarean sections: This variable was the average number of caesareans per month over the last six months as a percentage of all births over last six months in each facility.
8. Cost: Private obstetricians reported the minimum and maximum amount they charged for a vaginal or a caesarean birth. This was averaged to arrive at costs at each private facility for these services.

### Analysis

We conducted descriptive analyses, specifically medians, interquartile ranges and proportions to describe public and private obstetric facility characteristics. Bivariate analyses including Chi-square and Wilcoxon-Mann Whitney tests were used to compare characteristics between CY participant and non-participant facilities.

As CY participation was not rare (>40%), we created a Poisson Regression model with robust 95% confidence intervals to estimate prevalence ratios of facility characteristics that would predict CY participation [35,36]. Characteristics with p-values < 0.05 were included in the final model.

We used Research Electronic Data Capture (REDCap) for data entry and analyzed data using Stata (Version 12.0, StataCorp)

## Results

Childbirth services in the three districts had been provided by 300 facilities during the past year. They had attended to 53,896 births in the 6 months prior to the visit of the research team. While 135 facilities conducted less than 10 births per month, 165 facilities conducted ten or more births per month. This latter group accounted for 96% of the total births in the last six months in the three districts. Therefore we worked with this subgroup of facilities in the subsequent analyses presented. Seventy-one percent of these births occurred in private facilities, 31% in CY participant and 40% in CY non-participant facilities, while 29% occurred in public facilities. Births by caesareans took place predominantly in private facilities (Table 2).

**Table 2:** Distribution of numbers of facilities, self-reported childbirths and proportion of caesarean sections (CS) by facility categories.

Category of facility	Number of facilities	Births in last 6 months (%)	% Caesarean sections
Private CY participant	41	15935 (31)	10.5
Private CY non-participant	70	20383 (40)	21.4
Public	47	15376 (29)	2.6
Total	158	51694	11.0

**Fig 1:**
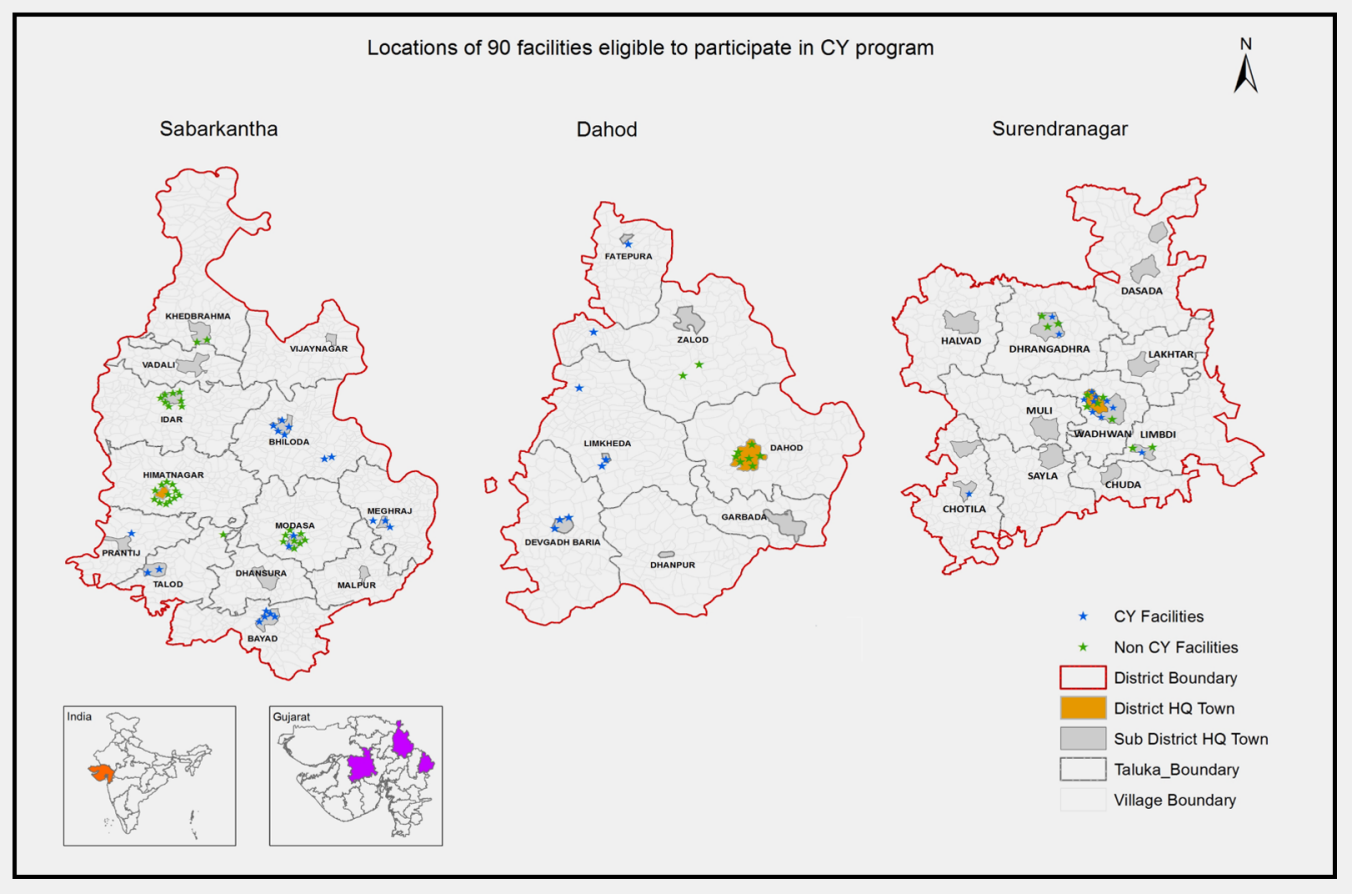
‘En masse’ participation/non-participation by eligible private providers in 9 out of 14 towns.

Of the 111 private facilities, 90 were eligible to participate in the CY program as per the criteria set by the state; however, only 40 did (44%). Our map revealed a pattern of CY participation. (Fig 1) CY participant facilities tended to be located in sub-district towns. In seven (Bhiloda, Meghraj, Bayad, Prantij, Talod, Devgadh Baria, Limkheda) out of 14 sub-district towns, all eligible facilities participated en masse in the program; while in two (Himmatnagar and Dahod) out of three district headquarter towns none of the private facilities participated. Three CY participant and four CY non-participant facilities did not consent to participate in the survey.

A descriptive analysis of the background characteristics of the participant and non-participant facilities has been presented in Table 3. Although both CY participant and non-participant facilities had been functioning since a median period of about 10 to 13 years, the median period of experience of obstetricians in participant facilities was significantly less by 7 years. While 40 CY facilities conducted a median of 35 vaginal and 6 caesarean births every month, 47 non-CY facilities conducted 41 vaginal and 11 caesarean births. (3 CY non-participant facilities did not allow us to review their records) The proportion of births by caesarean in CY (9%) and nonCY (23%) facilities in the last six months was significantly different. Costs for both vaginal and caesarean births were significantly different in CY and non-CY facilities.

**Table 3:** Comparison of characteristics of CY and NonCY facilities.

Facility characteristics (descriptive)	CY		NonCY)		Wilcoxon Mann Whitney test	
	(n = 40)		(n = 50)			
	Median		Median		p value	
	(IQR)		(IQR)			
Bed Strength	15		17		0.55	
	(10–23)		(12–22)			
Years of functioning	9.9		13		0.09	
	(2–17)		(7.4–20)			
Obstetrician’s years of experience	10		17		**0.01**	
	(3–30)		(10–24)			
Num of vaginal births recorded per month in last six months	35		41		0.90	
	(16–88)		(20–67)			
Num of caesarean births recorded per month in last six months	6		11		**0.00**	
	(1.5–10.5)		(6.7–18)			
Proportion of caesarean births in last six months	8.9		22.6		**0.00**	
	(2.8–23.9)		(12.4–37)			
Costs (Rs)						
Normal Delivery Costs						
Median	2500		3000		**0.005**	
Cost range	225–4500		250–7500			
Caesarean Costs						
Median	8500		10000		**0.006**	
Cost range	600–12500		1000–30000			

A simple comparison of CY participant and non-participant facilities showed that CY facilities tended to be located in sub-district headquarter towns and function as general hospitals, providing out and in-patient care for men and children along with maternity care (Table 4). They performed a lower proportion of caesarean sections, were owned by less experienced obstetricians and were cheaper. When individually tested in a Poisson model, a facility possessing each of the following characteristics separately, a general facility type, a facility performing less than 20% of births by caesareans, a facility with an obstetrician with less than 10 years of experience and a facility that charged less than Rs 2500 for vaginal births, was nearly twice as likely to participate in the CY program as a facility not possessing any of these characteristics. In the adjusted Poisson model, none of these variables retained significance (see Table 4).

**Table 4:** Facility characteristics which predicted CY participation: bivariate and multivariate prevalence ratios using a poisson regression model.

Facility characteristics	CY participants	CY non-participants	Unadjusted		Adjusted	
			
	n (%)	n (%)	PR	95% CI	PR	95% CI
DistHQ / SubdistHQ						
Dist HQ town	8 (20.0)	21 (42.0)		Ref		-
Sub district HQ town	32 (80.0)	29 (58.0)	1.9	1.0–3.6	-	-
Facility Type						
Purely Maternity	23 (57.5)	42 (84.0)		Ref		Ref
General (Maternity with other)	17 (42.5)	8 (16.0)	**1.9**	**1.3–2.9**	1.2	0.8–2.0
Total Bed Strength						
<15	25 (62.5)	21 (42.0)	1.6	0.9–2.6	-	-
>15	15 (37.5)	29 (58.0)		Ref		
Ave # of Vaginal Births per month						
<40	23 (57.5)	23 (48.9)	1.2	0.7–1.9	-	-
>40	17 (42.5)	24 (51.1)		Ref		
Proportion of births by C-section per month*						
<20%	28 (70.0)	18 (38.3)	**2.1**	**1.2–3.5**	1.7	0.9–3.0
>20%	12 (30.0)	29 (61.7)		Ref		Ref
Obstetrician’s yrs of experience						
Less than 10 yrs	22 (55.0)	14 (28.0)	**1.9**	**1.2–3.1**	1.4	0.8–2.3
More than 10 yrs	17 (42.5)	36 (72.0)		Ref		Ref
Median cost of vaginal births						
Less than Rs. 2500	24 (61.5)	17 (34.7)	**1.8**	**1.1–3.0**	1.2	0.7–2.0
More than Rs. 2500	15 (38.5)	32 (65.3)		Ref		

*DLHS II (2002 to 2004), NFHS III (2005–06), and DLHS III (2007–2008) data have shown that the national average urban caesarean rates among all institutional births across all states is 21.9, 17.8, and 17.1% respectively [37,38].

## Discussion

Our study found that 44% of eligible facilities participated in the program. Private facility attributes of being general hospitals, run by younger, less-experienced obstetricians, performing fewer Caesarean sections, and being less expensive were independently associated with likelihood of CY participation. However, this association did not persist in the multivariable model.

Participating facilities tended to be general hospitals. These hospitals tended to provide a broad range of services to children and men from the surrounding areas, not just maternity services. The shortage of health workers in more rural parts of the country has been well-documented. Qualified private providers are reluctant to work there and the public sector is unable to attract and adequately staff rural health facilities [39]. It is possible that a qualified specialist’s willingness to provide general services is indicative of the private provider’s long term commitment to the site of his practice and his urgency to establish his practice quicker by attracting a wider client base through participation in the CY program. This broader range of care provision by CY facilities can be used to mutual advantage by the state when designing PPPs in future since general facilities can provide a wider care package to the entire household.

We found that facilities owned by obstetricians new to private practice, tended to partner with the CY program. This supports the findings of the qualitative study of CY practitioners in the same districts [40], conducted under the same (MATIND) project which had reported that younger obstetricians in the process of establishing their private practices participated in the CY program to quickly “get name and fame”.

An associated finding in our study was that the median costs of both vaginal and caesarean births were significantly lower in CY participant compared to non-participant facilities. These findings were resonated in the qualitative study about CY where private obstetricians reported that market costs in sub-district towns was similar to the CY compensation package. In district towns, not only were routine charges well above the CY package, some obstetricians also perceived CY participation as ‘going down market’ making them less attractive to the wealthier clientele [40]. Similar to CY program, was the MAMTA program implemented in the capital city (Delhi) [41]. Almost 2/3rds of the private partners had reported their intention to leave the program because the compensation was lower than the real costs [42]. This may indicate that private obstetricians who charge less for their services tend to partner with the PPPs.

CY participant facilities performed significantly fewer caesarean sections than non-participants, though whether this characteristic was a precursor or a result of their participation in the CY program was not discernible due to the cross-sectional study design. This finding assumes significance in light of the fact that evidence from private practices in India and from other middle and low income countries such as Mexico, China, Lebanon, Egypt, Tunisia, Turkey and Dominican Republic [43–48]have shown an increasing rate of medical interventions (caesareans and induction) during the intrapartum period.

Although our study across three districts did not establish a significant difference between the characteristics of facilities that might predict participation in the multivariable model, it is possible that our sample was too small to detect heterogeneity within the groups. Based on the bivariate model and the map, it is possible that the more remote location in sub-district towns and newness of practices could account for the significant association of individual predictors like smaller hospitals, lower charges, lesser caesarean sections and the broad general services (not just maternity) provided by these facilities with CY participation.

### CY in the context of Public-Private-Partnerships (PPPs) for child birth services

PPPs have improved access to maternal services in many low and middle income countries [19] and have been recommended as an essential strategy to attain UHC [2,9]. Among the 5 countries that have instituted large scale PPPs for childbirth services, only Armenia’s program was universal (3 million population) [20].

In Kenya, Uganda, Bangladesh and Nepal, PPPs were targeted at poor populations of sizes varying from 3 million to 27 million [18]. Of these too, only Kenya and Uganda partnered with a reasonable number of for-profit private providers, 34 and 47 respectively [49,50]. Literature reveals that within India too, 5 states have implemented statewide PPPs targeted at poor populations for childbirth services, with 36 to over 200 for-profit private providers; but none of these lasted more than 2–3 years. Although none of these partnerships have been rigorously evaluated, most of them led to an increase in institutional deliveries as well as a rise in caesarean sections [20,42,51]. The urban location of private providers limited the reach of these programs. At least two of them reported that provider remuneration needed to be raised [42].

In comparison, Gujarat’s CY program was targeted at vulnerable households which constitute approximately 40% (24 million) of the state’s population. It has partnered with 350 to 850 qualified obstetricians for over a decade now, and more than a million deliveries have occurred under the scheme [23]. The remuneration package has been raised over the years but, due to its bulk-purchase character, has always been lower than the prevalent market rates (Table 3). Past assessments of the CY program have shown varying results; both beneficial effects such as reduction in maternal and neonatal mortality among the beneficiaries [26,52] and detrimental practices like participating private facilities treating only low-risk, uncomplicated cases, and referring the more complicated cases to public hospitals [53].One multivariate difference-in-difference study showed that, the CY program had no effect on institutional deliveries, maternal morbidity or out-of-pocket expenditures [54]. But it examined these outcomes after only one year of roll-out of the program in 5 pilot districts known to be underdeveloped [55]. A recent analysis of secondary data from the department of health showed that although the CY program did not lead to a rise in institutional deliveries in the targeted vulnerable group, it did result in a rise in caesarean rates. But this rise has been only 6% (compared to the prevalent rate of 18% in the private sector [37,38]) which is more in agreement with the WHO recommended range of 1–5% for ideal maternal outcomes [56,57]. The authors argued that this may indicate increased access to comprehensive EmOC care in the vulnerable population as a result of the CY program. The CY partnership contracts were designed to pay for EmOC intervention in only 15 out of 100 births, in accordance with indicator 3 of WHO/UNICEF/UNFPA guidelines [33]. This may have deterred unnecessary medical interventions and led to the significantly lower proportion of caesarean sections in CY facilities in our sample. But it is also possible that deserving cases were shifted out of the CY program. Thus the effectiveness of a CY-type payment package design to limit caesareans rates still needs to be investigated, because this could serve as a strategy for expanding PPPs in the future for Universal Health Coverage.

The same secondary analysis also showed that over the last decade, CY benefits have reached only one-third of the target population Table 1, [56]. One of the reasons for this could be the wide temporal and spatial variation of CY participation over the last decade in our study districts as shown by another study [58]. Even though, as per this study, the availability of EmOC care was not much affected across the districts, the fluctuations in local availability could be causing beneficiaries to drop-off locally.

However such variation is not unexpected and underlines the challenges that PPPs pose to achieve a fair distribution of free EmOC provision, even in areas where private obstetricians are abundantly available. We found that private obstetricians’ participation at the time of our survey was a group phenomenon (Fig 1). District headquarter towns had 20 to 30 private obstetricians who collectively stayed out of the CY program. While in smaller towns with less than 10 obstetricians, all of them tended to participate in the CY program. The study was unable to capture the reason for this group behavior of private practices. However, the objective of en masse participation in or out of the partnership can only be to level out any competitive edge within a town that would result from such participation.

The private sector in India is large and will continue to expand rapidly [59] PPPs are essential to achieving universal health coverage for India [40].The Universal Health Coverage plan for India needs to develop a framework for partnerships with the private sector in variable circumstances across the country. Experts argue that in the Indian setting, bureaucratic approaches to implementing regulations for health care delivery have failed. We now need to implement a market-oriented approach through collaborative mechanisms that enhance accountability [60]. Enhancing implementation of PPPs requires multi-sectorial inputs and rigorous implementation science which need to be built into public health governance [48]. The state must aim to produce effective health managers and leaders who can provide stewardship to PPPs for eg. control caesarean rates, design remuneration packages as per need, deal with collective phenomena like en masse participation or non-participation in the program, ensure geographic spread of participant facilities, as well as create trusting relations among providers, local regulators and the public.

### Strengths and limitations

This study is among the very few that explore details about PPPs and private sector maternity care in less developed areas of the world. Although we surveyed across a large geographic area, our yield of facilities eligible to participate in the CY program was only 90. A larger sample across more districts may have improved validity of our multivariable model, yielding a better explanatory power of predictor characteristics of facilities. Our final analysis for characteristics of CY participation excluded all private facilities with <10 births/ month. However, less than 5% of births occur in such facilities and therefore our results are representative of the majority of childbirths in the private sector. The generalizability of this study is limited to the more prosperous western and southern states of India which have comparable levels of urbanization and large numbers of qualified private providers [61].

## Conclusions

The CY partnership is a unique example of a PPP for maternity care, operating at scale for over a decade now. Our study provides clues to some gaps in the CY program that may be addressed to improve CY partnerships—attracting relatively newer obstetricians and general hospitals, tailoring remuneration packages to address need and remoteness, and minimizing spatial and temporal variations in partnerships. The government could now strategize the program and build it further around these characteristics. Maximizing the effectiveness of such partnerships requires that the state have strong managerial and analytical capacity to steer its course consistently over time.

## Supporting information

S1 File HR dataset. (DTA)

S2 File Facility dataset. (DTA)

S3 File Codebook. (DOCX)

S4 File Data cleaning and basic analysis stata code. (DO)

S5 File Descriptive analysis stata code. (DO)

S6 File Poisson analysis stata code. (DO)
