# Supplementary material for: Characteristics of private partners in Chiranjeevi Yojana, a public-private-partnership to promote institutional births in Gujarat, India – Lessons for universal health coverage
Source: PLoS One. 2017 Oct 17;12(10):e0185739. doi: 10.1371/journal.pone.0185739 (PMC5644975; doi:10.1371/journal.pone.0185739)
Supplement: S3 File — (DOCX) [file pone.0185739.s003.docx]

Form 1 – Primary information of all facilities

Form 2a – filled in facilities that hadn’t conducted in any deliveries in the last one year, but had the potential to conduct, i.e. they were mostly government subcentres which were equipped and “staffed” but not conducting deliveries. Variables collected in this from start with f2a…

Form 2bp – Private facilities, performing deliveries, but low load i.e. less than 10 deliveries per month over last 3 months (derived from form 1) Variables collected in this from start with f2bps1q11 (means form 2bp, section 1, question 1.1)

Form 2bg – Public facilities, performing deliveries, but low load i.e. less than 10 deliveries per month over last 3 months (derived from form 1) Variables collected in this form start with f2bgs1q11 (means form 2bg, section 1, question 1.1)

Form 2cp – Private facilities, performing deliveries, high load i.e. more than 10 deliveries per month over last 3 months (derived from form 1) Variables collected in this form start with f2cps1q11 (means form 2cp, section 1, question 1.1)

Form 2cg – Public facilities, performing deliveries, high load i.e. more than 10 deliveries per month over last 3 months (derived from form 1) Variables collected in this from start with f2cgs6q31 (means form 2cg, section 6, question 3.1)

Forms 2bg, 2bp, 2cg and 2cp had HR sections which were fed separately into RedCap.

Form 3 – was for assessment of signal functions

Data was entered into RedCap. We used Stata to analyse the data. All variable names and labels can be clearly visualized in Stata.

| -1 | Not available |
| --- | --- |
| -2 | Not answered |
| -3 | Not asked |
| -4 | Not mentioned (in records) |
| -5 | Not observed |
| -6 | Not clear |
| -7 | New question |
| -8 | Don’t know |
| -9 | Not applicable |
| -10 | Not filled |
| -11 | Amount unknown (money) |
| -12 | New category |
| -33 | Refused to answer |
| 99 | Missing |
